# Central nervous system stimulants in recreational and medical use

**DOI:** 10.1017/S1092852925100357

**Published:** 2025-07-14

**Authors:** Seetal Dodd, Laura Ospina-Pinillos, John S. Markowitz

**Affiliations:** 1 IMPACT—The Institute for Mental and Physical Health and Clinical Translation, Deakin University, Geelong, VIC, Australia; 2 Centre for Youth Mental Health, The University of Melbourne, Parkville, VIC, Australia; 3 Centre for Youth Mental Health, Barwon Health, Geelong, VIC, Australia; 4 Department of Psychiatry and Mental Health, Faculty of Medicine, Pontificia Universidad Javeriana, Bogota, Colombia; 5 Department of Pharmacotherapy and Translational Research, College of Pharmacy, University of Florida, Gainesville, FL, USA

**Keywords:** Stimulant, central nervous system, attention-deficit hyperactivity disorder (ADHD), drug abuse, amphetamine, catecholamine releasing agents

## Abstract

Stimulants that act on the central nervous system have been used since antiquity for ritual and other uses. Organic chemistry techniques, especially those developed in Germany in the late 1800s, resulted in the isolation and structural determination of several important stimulants. Synthetic pathways for amphetamine and related stimulants were developed in the first half of the 19th century, and these new drugs were widely marketed. Awareness of abuse potential emerged soon after but was contested. Stimulants have been used to counteract fatigue and promote wakefulness during military operations, as well as to treat sleep disorders, since the 1930s. Methylphenidate was approved to treat children with behavioral problems in 1962, predating the recognition of attention deficit hyperactivity disorder (ADHD). Stimulant abuse became a political concern in the post-war period, initially with the use of “pep-pills” by long-haul truck drivers and later as drug dealing became common in night clubs, with new laws limiting availability passed in the early 1960s. They have also been used to increase athletic and cognitive performance. Stimulants are still first-line therapies for ADHD and some sleep disorders; however, newer-generation drugs have been developed with better safety profiles and lower abuse potential. Illicit stimulant use continues to be common in many countries.

## Introduction

The psychostimulant class of drugs includes several chemical subclasses populated by derivatives of benzoylecgonine, phenethylamine, or aminoaryloxazoline. Psychostimulant is a name assigned to a large range of drugs that act on the central nervous system. In this paper, psychostimulant and stimulant will be used interchangeably. These agents produce a stimulatory effect on the central nervous system (CNS) and variably modulate the levels and activity of the major monoamine neurotransmitters dopamine (DA), norepinephrine (NE), and, to a lesser degree, serotonin. The differing degrees to which a specific agent affects these neurotransmitters determine the psychostimulant properties attributed to individual agents. Typical acute clinical effects may include, but are not limited to, heightened alertness, alertness increased energy, arousal, excitation, and elevated mood. Dose-dependent increases in pulse rate and blood pressure are also frequently noted. Other effects, such decreased appetite and improved cognitive performance, are also attributed to most stimulants. Stimulant drugs include drugs with mild effects at typical doses or exposures, such as caffeine and nicotine, and drugs that can be categorized elsewhere, such as 3–4-ethylenedioxymethamphetamine (MDMA), which shares properties as well as structural similarities to both a stimulant and a hallucinogen. Stimulants may be naturally occurring or synthetic, or synthetic drugs derived from molecules that are naturally occurring. Provided here is a brief historical overview of CNS stimulants followed by a bit greater focus and detail on amphetamine like drugs.

Archaeological evidence for the use of naturally occurring stimulants has been discovered in Peru, with the consumption of coca leaves^1^ dating to 8000 years ago, and tobacco has been identified at a site in the USA from approximately 12 300 years ago.^2^ At the time of the Spanish arrival, tobacco and coca leaves were used for ritual and general purposes. Describing use as recreational in indigenous communities is fraught, as it would not have been understood in that context. Chewing coca leaves was and is still used by indigenous workers in cold, harsh conditions and for the alleviation of fatigue, hunger, and thirst. Spanish colonizers who enslaved indigenous workers permitted them to chew coca leaves while working, claiming that it made them more affable and disposed to do perilous tasks.^3^ Elsewhere, the psychostimulant khat (or qat) was recorded being used as early as the 14th century^4^ and is currently commonly consumed in East Africa and the Arabian Peninsula. Of note for tobacco, coca leaves, and khat, their use within traditional communities is viewed as culturally normative and is stigmatized, or subject to restrictions or prohibition, only outside of their community.

**Figure 1.**
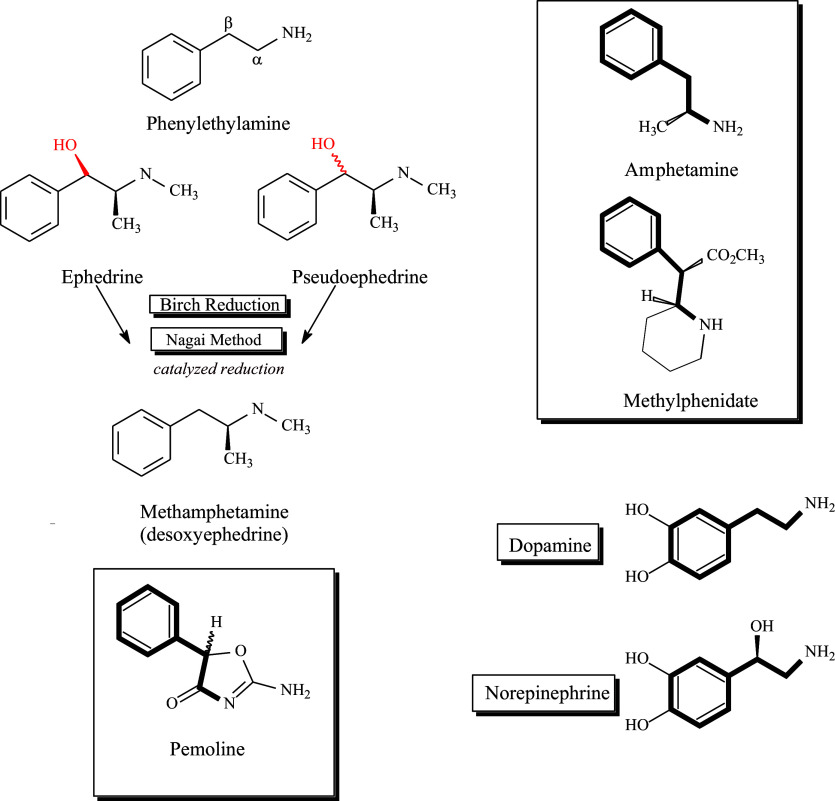
Structural relationships between phenethylamine, *Ephedra* alkaloids, monoamine neurotransmitters, and major psychostimulants.

Ephedrine and pseudoephedrine (Figure 1) occur naturally in plants of the genus *Ephedra* and were first isolated in 1885 by the Japanese organic chemist Nagai Nagayoshi.^5^ Herbal *Ephedra* was first documented in traditional Chinese medicine during the Han dynasty and is known as Ma huang and claimed to have been used in Chinese and Indian folk remedies for over 5000 years.^6^ In 1893, Nagayoshi synthesized methamphetamine using ephedrine as the precursor.^7^ Amphetamine, a contraction of the chemical name alpha-methylphenethylamine, was first reported in 1887 in a Berlin doctoral thesis of precursor derivatives by the Romanian chemist Lazăr Edeleanu.^8^ Later and independently, amphetamine was re-discovered by Barger and Dale in 1910 and synthesized by Alles in 1929, who was attempting to create a decongestant and bronchodilator as a synthetic alternative to ephedrine. Alles and others studied responses to amphetamine in animals and conducted clinical studies demonstrating its psychoactive properties.^9^ Soon thereafter, the pharmaceutical company Smith, Kline, and French (SKF) conducted further studies of amphetamine free base and marketed it as the Benzedrine nasal inhaler.

Registered under the trade name Benzedrine, racemic (*dl*) amphetamine was available over-the-counter (OTC) in in the early 1930s a nasal inhaler to treat nasal congestion and as a bronchodilator for asthma. Each small aluminium Benzedrine inhaler tube contained benzylmethyl carbinamine (racemic amphetamine base) 0.325 gm, oil of lavender, 0.097 gm, and menthol 0.032 gm. In the late 1930s, SKF also began the marketing of Benzedrine tablets (as racemic amphetamine sulfate) as well as single and more potent *d*-isomer marketed under the trade name of Dexedrine®.^10^ In 1943, the N-methylated derivative of amphetamine, *d*-methamphetamine, received FDA approval. Methamphetamine has a more potent effect on the dopamine transporter (DAT) compared to amphetamine, produces greater cardiovascular effects, and has a longer half-life. Many are surprised to learn that methamphetamine is still available for prescriptive use in 5 mg immediate-release tablets in the US. It presently carries an indication for the treatment of ADHD as well as exogenous obesity. Marketed as Desoxyn®, this name is derived from the chemical name desoxyephedrine, or dehydroxylated ephedrine (Markowitz and Patrick 2017).

Recognized patterns of abuse led to a change to prescription-only availability in 1937. Furthermore, beyond the initial characterization of amphetamine pharmacodynamics, investigations in the mid- to late 1930s began to identify pharmacodynamic-pharmacokinetic relationships that accounted for individual differences in response and individualized dosing in achieving optimal benefits.^11^ Additionally, a number of investigations assessed the different dosing schedules and frequency,^12^ and established when optimum effects occurred after administration^13^ and likely reflected attainment of time to maximum plasma concentrations (*T*_max_). Later, as adequate analytical methodologies were developed, fundamental pharmacokinetic studies were established for amphetamine and its respective isomers.^14^ Later racemic amphetamine became indicated for narcolepsy and alertness and available as 10 mg oral tablets. It was widely distributed to soldiers, initially in the Spanish Civil War, then on both allied and axis sides of the Second World War and remains currently in military use.^15^

Phenethylamine is a natural monoamine containing a phenyl ring connected to an amino group (–NH2) separated by a two-carbon chain that serves as the basic pharmacophore for a number of neurotransmitters as well as psychostimulant agents (Figure 1). It is also a naturally occurring compound found in a number of species and produces stimulant effects in its own right. Seemingly minor structural modifications can result in profound changes in CNS activity. Indeed, the simple attachment of a methyl group (–CH3) to the β carbon of phenethylamine yields amphetamine (phenylisopropylamine).

The main alkaloids present in *Ephedra* are the physiologically active diastereomeric pairs (−)-ephedrine and (+)-pseudoephedrine; (−)-methylephedrine and (+)-methylpseudoephedrine; and (−)-norephedrine and (+)-norpseudoephedrine (Figure 1). Of these, the secondary amine pair, (−)-ephedrine and (+)-pseudoephedrine. Ephedrine and pseudoephedrine are marketed in various dosage formulations and in combination with other medicinal agents and are employed as nasal decongestants or bronchodilators. Both compounds are known to serve as precursor compounds in the clandestine synthesis of methamphetamine (desoxyephedrine) by what are known as the “Birch Reduction” or Nagai Method” (Figure 1) and accordingly, in the US, ephedrine is no longer legally available for sale in the US as a single entity available for purchase and pseudoephedrine products are kept behind the pharmacy counter where individual purchases are logged and limited in amounts and frequency of purchase. Given their structural similarities to the *Ephedra* alkaloids, it is not completely surprising that both amphetamine and methamphetamine themselves as well as other amphetamine derivatives have been found elsewhere in the botanical world among some members of the *Acacia* species native to the Southwestern US and Northeastern Mexico.^16^

A number of analogues of amphetamine have been synthesized since the pioneering days of organic chemistry, and many of these analogues have stimulant properties. In addition, many of these molecules are racemic, and enantiomers have been separated. Dextroamphetamine is more potent than levoamphetamine and was first marketed in 1937 as Dexedrine. Amphetamine products have been sold under various trade names and formulated as base, salts, and various proportions of the two enantiomers. Indications for use expanded to well over 30 indications, including schizophrenia, opiate addiction, motion-sickness, radiation sickness, infant cerebral palsy, and persistent hiccups.^15^

Early use of amphetamine and related stimulants was not as stigmatized as other drugs, such as cannabis and opium, for various reasons. Harms and addictive properties emerged did not become well known until these drugs were in wide use. These drugs were prescribed by doctors and given to patients from mainstream European and American society, as well as to military personnel. In contrast, stigmatization of cannabis and opium was increased by racist associations with African descendant and Chinese populations. Reports of adverse effects of amphetamine use emerged as early as 1938, with two cases of psychosis reported after treatment. However, it was not possible to determine from these case reports if amphetamine had caused the psychosis, or if a latent psychosis was being unmasked.^15^ Case reports of addiction began to emerge but were similarly contested with the addictive properties of amphetamine only being fully recognized in the 1960s. Central nervous system neurotoxicity was also recognized in the 1960s. Currently, recreational stimulant use is highly stigmatized and popularly associated with a criminal, deviant, and dangerous stereotype.^17^

Throughout history, stimulants have been used to enhance physical and cognitive performance, from ancient athletes to modern professionals, despite the health risks and occasional fatalities such as the death of cyclist Tom Simpson in 1967. Despite regulatory efforts, stimulant misuse remains prevalent in sports, with stimulants ranking as the second most commonly detected banned substance in professional athletes. Misuse is also widespread among college and high school students in the US, where lifetime nonmedical use of stimulants ranges widely and is often motivated by academic pressures. Students report that stimulants are easy to obtain on campuses, and even those diagnosed with ADHD sometimes misuse their prescriptions. Common motivations include improving study focus, staying awake, and, less frequently, recreational use.^18^ Stimulant use has also been reported in professional environments such as Silicon Valley and among software developers, where studies have found prescription stimulants to be among the most commonly used substances to enhance productivity.^19^ At this writing, there are at least a dozen different amphetamine formulations on the US market approved for ADHD, which are different amounts or ratios of the amphetamine isomers and salts, or singular *d*-amphetamine incorporated into various pharmaceutical dosing technologies with differing release profiles. Additionally, recognizing the abuse potential of amphetamine by oral, intranasal, and intravenous routes the prodrug lisdexamfetamine (Vyvanse®) was developed and FDA approved in 2007. This formulation is comprised of an L-lysine amino acid covalently bonded to *d*-amphetamine. The intact prodrug has no CNS activity until it is metabolized by peptidases after oral administration, which slowly release *d*-amphetamine throughout the day. Thus, intranasal and intravenous routes produce little to no activity, discouraging abuse, and the slower release rate with the intended oral route produces a less desirable effect in stimulant abusers relative to shorter-acting immediate release amphetamine formulations.

The stimulant methylphenidate, α-phenyl-2-piperidine-acetic acid methyl ester (Figure 1) was first synthesized in 1944 by Leandro Panizzon, a chemist working for Ciba-Geigy (later Novartis) in Basel, Switzerland. Said to have been named “Ritalin” after Panizzon’s wife Rita, who used it to elevate her blood pressure while playing tennis, methylphenidate was initially indicated for depression, narcolepsy, lethargy, and barbiturate overdose.^20^ Unlike the single chiral center in amphetamine, there are two chiral carbon atoms within the methylphenidate molecule. Thus, a total of four isomers are possible. Some initial formulations contained a mixture of all four of these isomers (2 *erythr*o and 2 threo). However, the *erythro* isomers were later removed from the formulation due to some question of their CNS activity and potential association with greater side effects. Essentially all commercial methylphenidate formulations available today for prescriptive use are racemic (50:50) mixtures of *dl*-threo-methylphenidate. However, a few enantiopure (*d*-threo-[+]-MPH, dexmethylphenidate) products are available. It should be noted that essentially all of the CNS activity of methylphenidate resides in the *d*-isomer.^21^

In general terms, methylphenidate can be regarded as a noncompetative reuptake inhibitor of DAT and NET, whereas amphetamine is a monoamine releasing agent that will competitively inhibit DAT and NET at lower doses and inhibit VMAT2 at higher doses.^22^

Modafinil^23^ and pemoline^24^ are stimulant drugs that are not structurally related to amphetamine, methylphenidate, or naturally occurring stimulants. Modafinil and pemoline have a lower risk of abuse and addiction than amphetamine or methylphenidate. Their mechanisms of action have not been fully described.

## Medical use of stimulant medication

### Attention-deficit/hyperactivity disorder (ADHD)

Currently, stimulants are best known as the first-line treatment for attention-deficit/hyperactivity disorder (ADHD).^25^ However, when stimulants first began to be widely marketed in the 1930s and 1940s, ADHD as a diagnosis did not exist. Descriptions of children and adolescents with core symptoms of ADHD, excessive motor activity, inattention, and impulsiveness, have been described in literature dating before the modern era.^26^ However, attitudes toward childhood have changed remarkably in the modern period, suggesting that modern concepts of child and adolescent mental illness would have been incongruent with past attitudes. Childhood was not well understood in the past, where child labor and harsh discipline could be justified by erroneous beliefs. Conduct that was criminal or “immoral” was a major concern when considering child behavior. Nevertheless, early recognition of age-related inattention as a morbidity was described by Alexander Crichton in 1798.^26^ Descriptions of children being distracted, fidgety, overactive, noisy, or disobedient may have described children that we now recognize as ADHD, but these were not viewed as symptoms of an illness to be treated. Infant hyperkinetic disease, an early medical description similar to ADHD, was described in 1932.^26^ Hyperkinetic reaction of childhood was first included in the Diagnostic and Statistical Manual of Mental Disorders (DSM)-II of 1968,^27^ changing to attention-deficit disorder (ADD) in the DSM-III of 1980,^28^ and ADHD in the 1987 revision of the DSM-III.^29^ From the 1990s, in later editions of the DSM, ADHD was recognized as a chronic disorder in adults that does not necessarily disappear with childhood.

In 1937, the physician Charles Bradley used racemic amphetamine (Benzedrine) to treat children with behavioral difficulties at a psychiatric hospital who suffered from headaches following pneumoencephalography procedures. It was believed that amphetamine may stimulate the choroid plexus to produce cerebrospinal fluid. While showing no benefit for headache, a serendipitous finding of improved behavior and improved school performance was reported for 14 of 30 children by Bradley, the children’s teachers, and the children themselves.^30^ This observation became one of the earliest documented instances of amphetamines having a therapeutic effect on symptoms consistent with what is now recognized as ADHD. Amphetamine can be said to have been in continuous clinical use longer than any other pharmacological agent employed in modern psychiatry.^31^ Methylphenidate was approved by the FDA for the treatment of children with behavioral problems in 1962,^32^ with other stimulants and various formulations being approved since then.

Stimulant use for what many people view as behavioral problems received push back from the general public, some sectors of the medical community, and the government. By the 1970s a global anti-narcotics campaign was ubiquitous. The “War on Drugs” sought to prohibit all illicit psychoactive drug use, and by this time, abuse and addiction to stimulants were well known. ADHD was still viewed as a childhood disorder and was most commonly treated with non-pharmacological interventions. Sensationist media reports appeared of school children being drugged to control their behavior, leading to a Congressional Committee Hearing in 1970.^32^ Nevertheless, treatment of ADHD with methylphenidate or amphetamine formulations is considered first-line pharmacotherapy. These medications are generally well tolerated and have been shown to produce a positive clinical response in approximately 70%–80% of children diagnosed with ADHD.^33^ In 2011, pharmacotherapy treated two-thirds of cases of ADHD in the USA.^34^ Prescription stimulant usage doubled in the decade 2006 to 2016,^35^ suggesting that parents and clinicians have become more accepting of the benefits of stimulant treatment.

### Narcolepsy and sleep disorders

The following classic psychostimulants carry FDA-approved indications for Narcolepsy; mixed *d-* and *l*-amphetamine salts (Adderall®), racemic amphetamine (Evekeo®), and *d-*amphetamine and *dl*-methylphenidate (Ritalin®). Amphetamine has been used to treat narcolepsy since Benzedrine was first marketed. Modafinil and armodafinil as well as solriamfetol are indicated for narcolepsy. Modafinil also carries indications for obstructive sleep apnoea/hypopnoea syndrome and shift work sleep disorder.

### Obesity/binge eating

The effect of diminished appetite produced by psychostimulants has long been recognized and exploited for use in weight loss.^10^ Various amphetamine formulations were extensively prescribed for weight loss from the 1940s through the early 1970s, at which time there was an increasing recognition of both abuse and diversion of medications, and several actions were taken by Congress and the FDA to change controlled substances status and to even remove some products from the market. Nevertheless, methamphetamine tablets have remained on the market and carry an indication for the short-term treatment of exogenous obesity, and in 2014, racemic amphetamine sulphate tablets (essentially Benzedrine from the late 1930s) re-entered the clinical arena marketed under the proprietary name of Evekeo, carrying an indication for the treatment of ADHD in children >3 years of age but also in the short-term management of exogenous obesity. The *d*-amphetamine prodrug lisdexamfetamine (Vyvanse®) is FDA approved to treat moderate to severe binge eating disorder in adults. The related compound phentermine (Adipex® and others), a derivative of amphetamine, is currently marketed for weight loss as well as the phentermine-topiramate (Qsymia) combination.^26^

### Augmentation in treatment-resistant depression/fatigue and sleepiness symptoms related to depression

Stimulant medications have been explored as augmentation agents in treatment-resistant depression, particularly when symptoms, such as fatigue, sleepiness, and apathy persist despite standard treatment.^36^^–^^38^ Agents like methylphenidate and modafinil have shown benefits in improving energy and mood in selected patients. A 2023 meta-analysis of 13 randomized controlled trials involving 2478 participants found that psychostimulant augmentation significantly reduced depressive symptom severity compared to placebo (SMD = −0.18; 95% CI [−0.36, −0.01]; *p* = 0.04), although remission rates were not significantly improved.^36^ While not first-line, stimulants may be appropriate in carefully chosen cases when other strategies have failed and low energy remains a major barrier to recovery. Methylphenidate is also sometimes employed in low dosages in elderly individuals as a means to improve apathy and mood.

### Non-medical treatments

#### Military use

There is a long, secretive, and controversial history of psychoactive drugs being used by the military to gain an edge against an enemy. Psychedelics, including lysergic acid diethylamide (LSD) and psilocybin, benzodiazepines, barbiturates, and the antimuscarinic scopolamine are all examples of drugs that have been trialed by the military. The military has tried to investigate if drugs could be used to make people divulge secrets or otherwise be manipulated. Stimulants have been and are still used to improve performance and maintain wakefulness. The secret nature of the military means that only limited information is available in the public domain, with historic use of stimulants more publicly known than current use.

Stimulants were first given to troops in combat in the Spanish civil war and usage became widespread in the second world war. German troops were issued with Pervitin (methamphetamine), Japanese troops were issued with Philopon (methamphetamine) and American and British troops were given Benzedrine (amphetamine). The German Wehrmacht considered Pervitin to be highly suited to methods of “blitzkrieg,” enabling tank crews and other mechanized forces to advance rapidly without regular rest and sleep, and issuing more than 35 million tablets by 1941. Allied sources described these troops as “heavily drugged, fearless and berserk,” suggesting that stimulant use had additional benefits to wakefulness, contributing to battlefield performance.^39^ On the allied side, 250–500 million Benzedrine doses were issued, with 15% of troops being regular users. Stimulant use increased performance in fatigued troops and were reported to increase aggressiveness, confidence, and self-esteem.^40^ Both allies and axis were aware of the risks of addiction and adverse effects with these drugs, but these considerations were largely ignored.

During the Gulf War in 2003, an incident occurred where American pilots bombed Canadian troops killing four soldiers. The use of Dexedrine (dextroamphetamine) by the US pilots was suggested to have contributed to the pilot error.^41^ Limited information is available on military use of stimulants, but it is believed to be ongoing at some level. Captagon (fenethylline) has been used widely in the Syrian civil war.^42^

#### Cognitive enhancers

Stimulant use was established from early trials to improve school performance in children with ADHD. Stimulants, including methamphetamine, have been used by non-ADHD students to promote wakefulness and enhance academic performance.^43^ Improvement was demonstrated to be greatest for individuals with low baseline performance, suggesting benefit to low-performing cohorts rather than benefit overall.^43^ Modafinil has been suggested to improve cognition where it is impaired, such as in schizophrenia, and to decrease fearful stimuli in healthy subjects, reducing anxiety and enhancing efficiency of prefrontal cognitive information processing.^23^ Corydrane, a mixture of amphetamine and aspirin marketed in France until its withdrawal in 1971, was popular among some French intellectuals, including Jean-Paul Sartre, who claimed that it assisted him with his writing.

Chronic methamphetamine use has been shown to cause persistent dopaminergic deficits, including loss of striatal DA and changes to gray and white matter density. Cognitive function is reduced in high-dose, regular methamphetamine users.^44^

#### Sports doping

Section S6 of the World Anti-Doping Agency 2025 international standard prohibited list prohibits all stimulants, including all optical isomers.^45^ Stimulants have been used by athletes to enhance performance by reducing tiredness and fatigue. Recreational drugs have been detected in routine screening of some high-profile athletes.

## Amphetamines

Amphetamine (1-phenylpropan-2-amine) is a racemic molecule owing to its single chiral carbon and is a mixture of dextro- (or *d*-) and levo- (or *l*-) isomers, with *d*-amphetamine being the more active isomer of the two. Pharmacologically active molecules in this group are predominantly stimulant and sympathomimetic drugs; however, not all molecules with a similar structure to amphetamine are pharmacologically active, and all molecules vary in potency. Some amphetamine structural analogues belong to other drug classes, including the monoamine oxidase inhibitors tranylcypromine and selegiline (also known as deprenyl). A further indication of the structural similarities of the molecules is that small amounts of *l-*methamphetamine and *l*-amphetamine are formed as metabolites of selegiline. Some amphetamine analogues are used for their sympathomimetic properties rather than their stimulant properties, for example, pholedrine and dioxethedrine.

The structural relationships between amphetamines; precursors, derivatives, analogues, and stereoisomers, and their neural substrates, DA, and NE, are shown in Figure 1. The structures included are not exhaustive but do include all medicinal amphetamines.

Amphetamines are viewed primarily as catecholamine-releasing agents. Through action facilitated by the inhibition of the vesicular monoamine transporter 2 (VMAT-2), amphetamine can induce DA and NE release from presynaptic vesicles, bind to presynaptic dopaminergic and noradrenergic neurons inducing monoamine release at the nerve terminal, and bind to the DAT and NE reuptake transporter (NET), reversing the direction of monoamine transport from the nerve terminal into the synaptic cleft.^46^^,^^47^

Amphetamines were heavily marketed in the 1930s and were very widely used in the Second World War, resulting in large numbers of people developing use disorders as well as a familiarity with these drugs. In the post-war period, amphetamines, known as “pep-pills” were well known to long-haul truck drivers.^48^ Concerns with amphetamine abuse led to their prohibition by 1962^49^; however, by that time they had already become well known in night clubs and an illicit trade had developed.^50^ International data from wastewater monitoring demonstrates that amphetamine and methamphetamine are among the most widely used illicit recreational drugs, although the rate of use is not evenly distributed and peaks in East and North–Central Europe, with methamphetamine peaking in the United States and Australia.^51^

## Phenidates

The psychostimulant methylphenidate remains the only “phenidate” medication approved for prescriptive use in the US and elsewhere. Although a plethora of new methylphenidate therapeutics have been approved by various regulatory agencies and made it into the marketplace for the treatment of ADHD since the 1960s, their differences are limited to the introduction of a variety of modified-release oral dosage forms as racemic mixtures and single isomer (*d*-methylphenidate) products and a transdermal patch. Similar to the scenario described above for the development of the *d*-amphetamine prodrug lisdexamfetamine as a less abusable dosage form, a prodrug of *d*-methylphenidate was developed called serdexmethylphenidate in hopes of receiving a lower scheduling status but having the same clinical benefits as methylphenidate. The prodrug was designed to be pharmacologically inactive until it is gradually metabolized in the lower intestinal tract, releasing *d*-methylphenidate. Its inactivity as a prodrug discourages abuse by the intranasal and iv routes, and the slower release rate of the oral formulation decreased the “likeability” by stimulant abusers in targeted studies. However, the slow release and onset of action that decreased the likeability of the drug in stimulant abusers appeared to be the same pharmacokinetic:pharmacodynamic profile that produced suboptimal performance in clinical trials for the treatment of ADHD—particularly in the first several hours following dosing. To resolve this issue, it was decided to add to the formulation a fraction of the immediate-release *d*-methylphenidate. Ultimately, the final formulation receiving FDA approval in 2021 (trade name Azstarys®) was a combination containing *both* the prodrug serdexmethylphenidate (70%) and immediate-release *d*-methylphenidate (30%), which provided more immediate clinical effects as well as coverage throughout the day.

**Figure 2.**
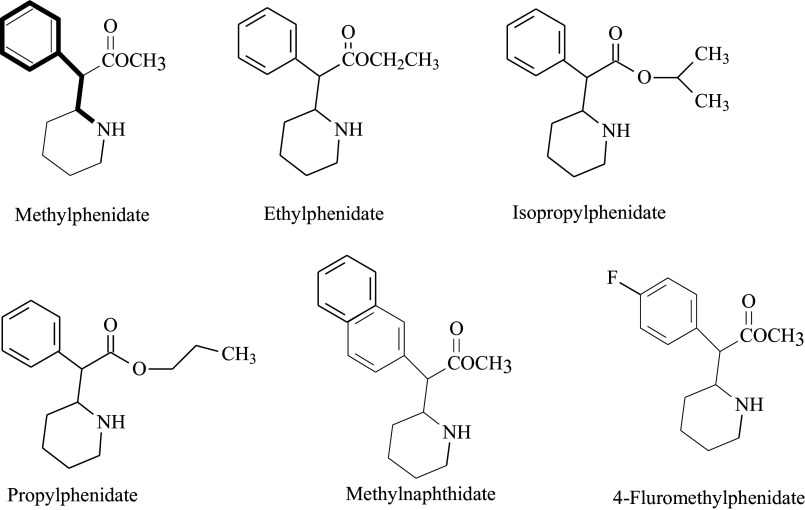
Examples of phenidates.

A number of other phenidate derivatives are in existence and can be found largely described in the new psychoactive substances (NPS) biomedical literature (Figure 2). NPS refers to a large and diverse group of substances of abuse not yet completely controlled by international drug conventions. A complete review of NPS is beyond the scope of the present chapter, but a few comments here on this phenomenon are useful. Many of the NPS substances were synthesized and patented many decades ago, but only recently have had their chemistry modified to produce new substances with similar subjective effects to established recreational drugs.^52^ These so-called “designer” or synthetic drugs that are frequently structural analogues of existing prescriptive controlled drugs or other known drugs of abuse. These compounds are specifically created to produce substances with similar or enhanced psychoactive effects to other controlled substances or illicit drugs.^53^ These structural changes to the compounds can make substance identification problematic and pose problems for toxicologists and emergency response personnel responding to NPS intoxication or overdose scenarios. Identification NPS has found their way into cryptomarkets for internet purchases. Phenidates in general are chemical analogues of methylphenidate.^54^^–^^56^ Portoghese and Malepeais (1961) initially reported on the synthesis and characteristics of a series of alkyl esters of methylphenidate. Among these were ethylphenidate, n-propylphenidate, and n-butylphenidate.

## Ethylphenidate

**Figure 3.**
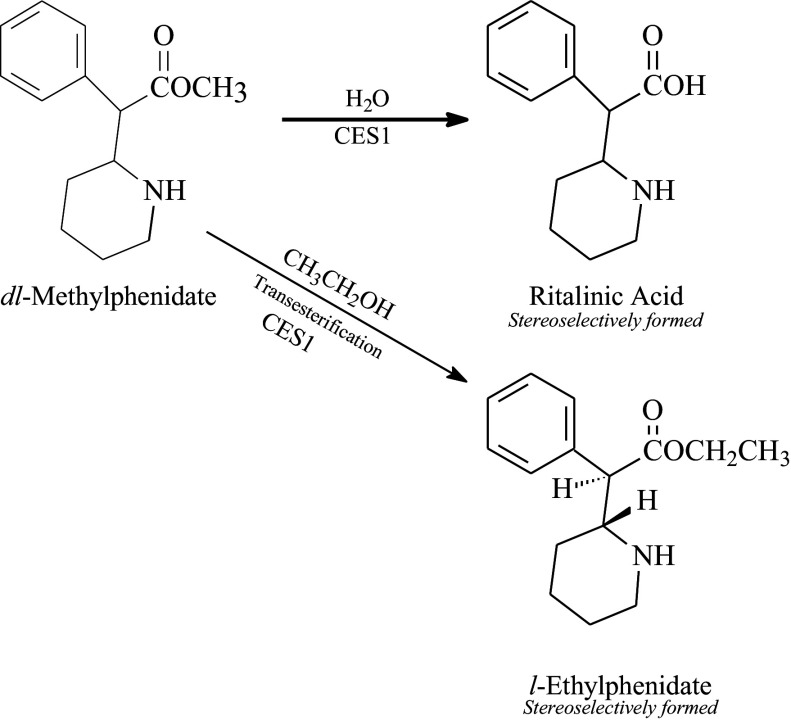
Ethylphenidate formation via transesterification with ethyl alcohol.

Ethylphenidate (EPH) is the ethyl acetate analogue of MPH (ethyl phenyl (2-piperidinyl) acetate, Figure 2). It is an amphetamine-like stimulant that inhibits both DAT and NET in the CNS. The affinity of EPH to DAT and NET is similar to those of MPH, but its affinity to NET and inhibition potency are substantially different, indicating qualitative differences between the pharmacodynamics of MPH and EPH.^57^ An additional curiosity regarding ethylphenidate is that it can also be found as a biotransformation product formed via a carboxylesterase 1 (CES1)-catalyzed transesterification reaction when ethanol is co-consumed with methylphenidate (Figure 3).^58^ The amount of the ethylphenidate metabolite formed endogenously by this reaction is fairly low and appears to proceed largely in a stereoselective process, being formed as *l*-ethylphenidate. However, large quantities of *dl*-ethylphenidate became available over the dark web and were marketed as a powder or a crystal and are frequently consumed by insufflation or injected intravenously.^59^ This is significant in that these dosing routes avoid the substantial first-pass effects that occur with orally administered methylphenidate, which limit its bioavailability to less than 25%. Intravenous use produces 100% bioavailability, and intranasal administration almost certainly provides greater exposure than oral dosing. Ethylphenidate is considered to be a highly abusable substance, and a number of fatalities have been reported.

## Isopropylphenidate

Isopropylphenidate (IPH) is the isopropyl acetate analogue of MPH (isopropyl phenyl(2-piperidinyl) acetate (Figure 2).^60^ It appeared on the cryptomarket in 2013 but was first discussed in the literature in 1961. IPH binds DAT and NET with an affinity similar to that of EPH.^60^

Beyond ethylphenidate, a number of other analogues of MPH have been produced through a simple extension of the carbon side chain (eg, isopropylphenidate, propylphenidate). Furthermore, substitution of the phenyl ring with a 1-naphthyl ring is a further modification producing CNS active derivatives, including ethylnaphthidate and methylnaphthidate. Other substituted “phenidates,” which appear to be potential substances of abuse with likely CNS stimulatory effects include propylphenidate, N-benzyl-ethylphenidate, methylmorphenate, 3,4-dichloromethylphenidate, 3,4-dichloroethylphenidate, 4-fluoroethylphenidate, 4-fluoromethylphenidate, and 4-methylmethylphenidate.

## Pemoline

Pemoline (2-imino-5-phenyl-4-oxazolidinone) was a third psychostimulant molecule available to treat ADHD that was originally approved by the US FDA in 1975. Marketed under the trade name Cylert®. Though less frequently prescribed than amphetamine and methylphenidate formulations and thought to have lower abuse potential, the drug was removed from the US market in 2005 due to accumulating reports of pemoline-associated hepatotoxicity. Although seemingly structurally dissimilar to amphetamine and methylphenidate, the shared phenethylamine structure, a common pharmacophore between it and the other stimulants, can be appreciated in bold (Figure 1). Pemoline is a presynaptic DA-releasing agent and re-uptake blocker. Pemoline was used to treat ADHD but was withdrawn from sale due to rare but serious cases of liver toxicity. Pemoline abuse has been reported.^61^

## Modafinil

**Figure 4.**
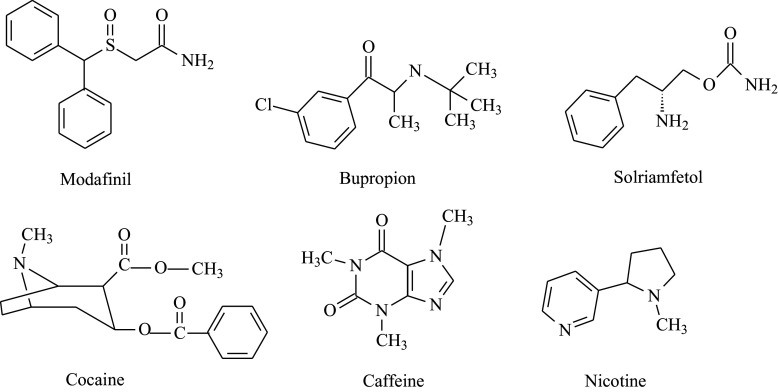
Miscellaneous examples of psychostimulants.

In 1974, while screening molecules to detect analgesic properties, French chemists R. Gombert and E. Assous from the pharmaceutical company L. Lafon Ltd. found that the molecule CRL 40028 was pharmacologically active.^62^ Studies found that CRL 40028 increased locomotor activity in mice, and this hyperactivity did not occur when CRL 40028 was given to mice that had been pre-treated with a blocker of α-adrenergic receptors.^63^ CRL 40028 was later named adrafinil. The mechanism of action of adrafinil as a stimulant is adrafinil has metabolites modafinil (CRL 40476) and an acid (CRL 40467). Modafinil (Figure 4) is further metabolized into a sulfone. Acid and sulfone metabolites are pharmacologically inactive. Armodafinil the *R*-enantiomer of modafinil,^64^ was approved by the US FDA in 2007 under the trade name Nuvigil® as an enantiopure formulation with approved indications to improve wakefulness in adult patients with excessive sleepiness associated with obstructive sleep apnea, narcolepsy, or shift work disorder (SWD).

Adrafinil and modafinil are racemic mixtures. Derivatives have been synthesized, including fluorinated derivates fladrafinil, flmodafinil, and modafiendz, as well as heterocyclic derivatives. For several derivatives, there is a paucity of published research, especially from human trials.

Among these drug analogues, modafinil has been the most thoroughly researched. Nevertheless, even for modafinil, the detailed mechanism of action is not fully known. Stimulant pharmaco-activity is prevented by blocker of α-adrenergic receptors, but modafinil has not been shown to be an α-adrenergic receptor agonist.^65^ Unlike amphetamines, modafinil is not a catecholamine-releasing agent.^66^ Modafinil and armodafinil bind to DAT, but not with the high binding affinity of other stimulants such as cocaine. Modafinil increases DA in the striatum and cortex.^66^ Armodafinil has a greater binding affinity for DAT than modafinil. Modafinil does not bind to presynaptic DA receptors.^66^ Modafinil does not bind to serotonin (SERT) or NE transporters (NET). The mechanism of action of modafinil as a stimulant is through DA and NE; however, modafinil has also been shown to influence gamma-amino butyric acid (GABA), serotonin (5HT), glutamate, histamine, and orexin/hypocretin.^66^

Modafinil is administered by oral dose and reaches a blood plasma concentration maximum (*C*_max_) after 2–4 h. The elimination half-life (t⅟_2_) is 12–15 h for the *R-*enantiomer armodafinil and 4–5 h for the *S*-enantiomer.^66^ Consequently, modafinil is not useful for immediate alertness, and armodafinil has benefits over modafinil for duration of action.

Modafinil and related drugs have a low potential for addiction or dependence, as they lack the pleasurable effects of classic stimulants. Cases of dependence have been reported, manifesting as dose escalation.^67^ The favorable safety profile of modafinil resulted in its use as the preferred military anti-fatigue drug, used currently by pilots.^68^ Modafinil is indicated for the treatment of sleep disorders, including narcolepsy, obstructive sleep apnea/hypopnea syndrome, and shift work sleep disorder. The use of modafinil to treat ADHD is off-label.

Controversially, modafinil and related drugs have been used as nootropic (cognitive enhancer) agents by students sitting exams^69^ and as a performance-enhancing drug in sports. Modafinil is included as a prohibited substance in the World Anti-Doping Agency (WADA) prohibited list for drugs in sport.^45^ Modafinil was shown to be superior to placebo for improvement to spatial working memory, planning, and decision making in non-sleep-deprived health volunteers,^70^ although benefits are at best modest.

## Bupropion

Bupropion, classified as an aminoketone antidepressant (Figure 4), is a mild stimulant due to DA and NE re-uptake inhibition. It was initially FDA-approved for the treatment of depression in 1989 and later as a smoking cessation aid.^71^ In 2022, a fixed combination of bupropion and the N-methyl D-aspartate (NMDA) receptor antagonist and sigma-1 receptor agonist, dextromethorphan, was approved under the trade name (Auvelity®) with an indication for the treatment of major depressive disorder in adults. This fixed combination appears to reduce depressive symptoms more quickly than bupropion alone. Bupropion has been evaluated for the treatment of ADHD but appears to offer only small reductions in symptoms for most patients. Bupropion abuse has been reported, including recreational ingestion, nasal insufflation of crushed tablets, and intravenous injection.^72^ Bupropion has emerged as a drug of abuse in prisons.^73^

## Solriamfetol

Solriamfetol (Figure 4) is a newer stimulant approved for excessive daytime sleepiness (hypersomnia) associated with narcolepsy and obstructive sleep apnea.^74^ A pilot trial randomized individuals (*N* = 60) to solriamfetol or placebo and found that solriamfetol was well tolerated and superior to placebo for several outcome measures,^75^ including clinical global impression and ADHD symptoms. Elsewhere, a case report described an ADHD patient who had responded poorly to atomoxetine and methylphenidate, who subsequently responded to solriamfetol treatment.^76^

Solriamfetol is a low potency, selective DA, and NE reuptake inhibitor. It is not associated with locomotor effects observed with higher potency stimulants.^77^

## Cocaine

Cocaine, a tropane alkaloid (Figure 4), is a powerful stimulant extracted from coca leaves. Use of coca leaves by indigenous communities in South America has a long history and cultural significance. Consumption of coca leaves and use of cocaine are in no way equivalent.^78^ The use of coca leaves is outside of the scope of this chapter. Cocaine was first extracted from coca leaves in Germany in the 1850s by Friedrich Gaedcke, who first isolated crystal in 1855,^79^ and Albert Niemann, who published an improved method for extracting cocaine in his PhD thesis published in 1860.^80^

The medicinal value of cocaine was soon investigated, first demonstrated for use as a topical anesthetic for eye surgery in 1884, the anesthetic properties of cocaine quickly became its greatest success as a medicinal agent,^81^ and it is still used today in solutions varying from 1% to 10% concentration on mucosal surfaces for ophthalmology and otolaryngology. Cocaine was also important in some pioneering research into local anesthesia, as a nerve blocking agent and forerunner for local anesthetics, including procaine and lidocaine.^82^

The stimulant properties of cocaine were also quickly recognized, and it was recommended for medical and non-medical use, often by physicians who had tried it on themselves or were regular users over some period of time. Sigmund Freud consumed cocaine and used it to treat his patients, publishing his work “Über Coca” in 1884, where he enthusiastically praised cocaine hydrochloride as a treatment for physical and neurological conditions.^83^ Over a period of years, Freud enthused over the potential for cocaine to treat addiction, including alcoholism and morphine abuse, but later accepted that cocaine also had abuse potential. His support for cocaine to treat melancholia lasted a bit longer, but his last published works regarding cocaine were in 1887.^83^

Cocaine has been marketed as a hay fever remedy^81^ and a treatment for headache, among many other uses.^3^ In addition, it has been an ingredient in Coca Cola^81^ and in Vin Mariani, famously endorsed by Pope Leo XIII.^84^

Reports of harm from cocaine use emerged from the 1880s, leading to restrictions on the importation, manufacture, and distribution of cocaine in the USA with the Harrison Narcotics Act in 1914.^3^ Currently, cocaine is estimated to cause 50 000 death per year worldwide.^3^ There is no current interest from the medical community in the stimulant properties of cocaine as a pharmacotherapy.

Stimulant effects of cocaine are due to inhibition of DAT and NET. Cocaine is also an antagonist of adrenergic receptors. Local anesthetic effects are due to inhibition of voltage-gated sodium channels blocking neuronal potentiation.^3^ Cocaine pharmacokinetics varies depending on the route of administration, with intravenous injection, nasal insufflation, and smoke inhalation all being common. Cocaine is extensively metabolized with two major metabolites, benzoylecgonine and ecgonine methyl ester, among 11 known metabolites.^3^

## Caffeine and nicotine

Caffeine and nicotine (Figure 4) are classified as mild stimulants. Caffeine is an antagonist of A1 and A2 adenosine receptors and has beneficial effects for wakefulness. Stimulant properties are through binding with adenosine receptors that have formed heteromers with DA receptors, modulating the activity of the DA receptors.^85^ There is limited evidence to suggest caffeine may have beneficial effects for some cancers, has anti-inflammatory effects, and may be neuroprotective.^86^ Nicotine binds to nicotinic acetylcholine receptors, activating the dopaminergic system.^87^ Nicotine is not suitable for medical use as it is highly addictive, other than nicotine replacement therapy for smokers. These two substances are frequently consumed together in the form of coffee and other beverages by tobacco smokers. Caffeine is reported to potentiate the reinforcing and stimulant effects of nicotine, which may make nicotine use more rewarding.^88^

## Discussion

Community attitudes and stigma toward recreational drug use have a history that varies from drug to drug. In the 1930s, at a time when cannabis, an arguably less harmful drug, was highly stigmatized and associated with African Americans and jazz music, amphetamine was associated with white society, intellectuals, brave soldiers, and increased work productivity. Stimulants were manufactured and marketed by reputable pharmaceutical companies, endorsed by the Pope, and consumed by intellectuals and authors, including Wystan Hugh Auden, Jean-Paul Sartre, Graham Greene, and Ayn Rand.^89^ Even in fiction, stimulant use was what the good guys did, such as Sherlocke Holmes.^83^ Stigmatization and a shift in community attitudes occurred when amphetamine use patterns shifted to become associated with lower social classes, truck drivers, and night clubs. Currently, stimulant use is highly stigmatized, with an Australian study finding high levels of stigma and discrimination toward methamphetamine users, including self-discrimination by users themselves. In a study of crystal methamphetamine users (*n* = 564) and non-users (*n* = 1544), 23.4% of users and 50.2% of non-users endorsed the statement “People who use ice are dangerous” and 50.7% of users and 71.2% of non-users endorsed the statement “I avoid people who use ice whenever possible.” Stigmatizing attitudes were greatest among females, those with less knowledge of crystal methamphetamine, and those living in regional locations.^17^

Currently, stimulants are first-line therapies as well as the most common therapies for ADHD. New treatments are emerging that are not stimulants, such as atomoxetine and viloxazine; however, the efficacy of amphetamines and phenidates has not been surpassed by the non-stimulant agents. There is a need for newer agents with better safety profiles and lower risk of addiction. Non-pharmacological therapies also have a role in treating ADHD and may be preferred by some patients and their parents, although currently stimulant therapy is having an increasing role in treating this indication, in spite of continuing stigma. Safer agents are also a focus of interest for the treatment of sleep disorders.

Non-medical and recreational use of stimulants varies depending on local availability and preferences and may change with time. Despite the stigmatization and illicit status of drug use and the well-documented personal and societal harms, stimulant use is commonplace in many countries. Rather than a prohibitionist, “War of Drugs” approach, a managed, harm-minimization approach may be more appropriate.

## Conclusion

Stimulant drugs are catecholamine-enhancing agents that have been used since antiquity and may increase work productivity, endurance, and performance for some tasks. Synthetic stimulants, such as the amphetamines, have a more recent history, originally as pharmaceutical products. Stimulants are first-line therapies for ADHD and are also used to treat some sleep disorders. Non-medical uses include maintaining wakefulness, especially for tasks that require alertness for long periods. Stimulants are among the most commonly used recreational drugs.
